# Editorial: Unravelling the Role of HERVs in Cancer: Insights and New Targets for Therapy

**DOI:** 10.3389/fonc.2022.874245

**Published:** 2022-03-14

**Authors:** Claudia Matteucci, Emanuela Balestrieri, Tara Patricia Hurst, Gkikas Magiorkinis, Reiner Strick

**Affiliations:** ^1^ Department of Experimental Medicine, University of Rome Tor Vergata, Rome, Italy; ^2^ Department of Life Sciences, School of Health Sciences, Birmingham City University, Birmingham, United Kingdom; ^3^ Department of Hygiene, Epidemiology and Medical Statistics, Medical School, National and Kapodistrian University of Athens, Athens, Greece; ^4^ Department of Gynaecology and Obstetrics, Laboratory for Molecular Medicine, University Hospital Erlangen, Erlangen, Germany

**Keywords:** genome instability, endogenous retroviruses, cancer therapy, cancer biomarker, oncogenic mechanisms

Current cancer therapy needs innovative approaches to identify new targets to overcome tumour aggressiveness, immunosuppression and drug resistance. In this scenario human endogenous retroviruses (HERVs) have been demonstrated to be potential causative elements or co-factors contributing to the onset and progression of cancer in humans. Upon initiation of this Research Topic, we sought contributions focusing on the role of HERVs driving tumourigenesis, as well as identifying HERVs as novel diagnostic and prognostic biomarkers, and targets for therapy.

In a review article, Kitsou et al. focused on the role of both exogenous and endogenous retroviruses in human cancers, highlighting how omics approaches have improved the understanding of the mechanisms underlying virus-mediated tumourigenesis. In addition, human-viral interactions at the level of anti-viral intrinsic mechanisms, and the participation of the virome in the structure and activity of the human flora were investigated. A comprehensive review concerning the endogenous counterpart, the multiplicity of HERVs’ roles in the pathogenesis of human malignancy have been exposed, detailing the underlying mechanisms, such as chromatin structure modification and imprinting, transcriptional and post transcriptional modulation, and the mechanism of cancer immune escape.

In their dynamic regulation, HERVs are important regulators of the immune response as well as key determinants of pluripotency in human embryonic stem cells, and have been associated with the cancer stemness phenotype (1, 2). In this regard, Glinsky focused on HERV transcription and regulation of human embryonal stem cells using 94,806 human-specific regulatory sequences, including 35,074 neuro-regulatory human-specific single-nucleotide changes, revealing stem cell-associated retroviral sequences (SCARS). These SCARS were hypothesized to be pivotal for stem cell differentiation, but SCARS overexpression in differentiation-defective cells can lead to malignant growth. SCARS regulate expression of a majority of cancer survival predictor genes and cancer driver genes, which is consistent with the hypothesis implicating their dysregulation in the pathogenesis of multiple types of human malignancies. In another research article, using the so-called LL-100 panel, which consists of RNA-sequencing gene expression data of cell lines with haematological cancers and stem cells, Engel et al. found 13 HERV-families differentially expressed. Interestingly, most of these HERV families were upregulated in hematopoietic stem cells, whereas only some HERV families were upregulated or downregulated in certain haematological cancers. More specifically, HERV-E1, ERV3-1, HERV-K HML-2, HERV-H and HERVH48-1 (ERV-Fb), were found higher expressed in stem cells. Recently, an essential role of HERV-H for the transcription of other genes in pluripotent stem cells *via* chromatin remodelling was shown (3). It will be important to elucidate, if these HERVs detected by Engel et al. show similar regulations. In another research article, neuroblastoma cell lines were analysed and various HERVs, like ERV3-1, HERV-E1, HERV-Fc2, HERV-K and HERV-W1 Env (Syncytin-1) were found to be transcriptionally activated (Wieland et al.). In addition, cultivating cells in a stem cell serum free medium, the authors showed overexpression of HERV-R (ERV3-1), HERV-E1 and HERV-Fc2 as well as of other genes, like the neuronal lncRNA MIAT, recalling the profound influence of microenvironmental changes on the regulation of the expression of HERVs.

The research article by Dolci et al. investigated the role of multiple HERVs, as well as Alu and LINE-1 elements, in colon cancer. The study interrogated not only transcription and protein expression but also examined the methylation status through pyrosequencing. Importantly the study provides further evidence for the common idea that there is global hypomethylation in tumours, where Alu, LINE-1, HERV-K (HML-2) and HERV-H elements show decreased methylation in tumour samples compared to adjacent normal tissues. Of these, the difference of methylation was only significant for LINE-1. Interestingly, the transcription of HERVs did not differ significantly between normal and tumour samples, but in contrast the protein expression of HERV-K Pol and Env was different. The former was found to be more intensely expressed in the adjacent normal than in the tumour samples, whereas the latter was only expressed in the tumour samples. Indeed, the authors propose a kinetic effect whereby HERV-K Pol may favour the initial stages of transformation through the enzymatic activity of the encoded reverse transcriptase. HERV-K Env, on the other hand, may be more important at the later stages, promoting tumour development.

The review article by Dervan et al. describes the upregulation of HERV-K Env as a feature of cancers such as of prostate, breast, melanoma and haematological malignancies. The review proposes to harness this upregulation for diagnostic and therapeutic purposes, such as the use of HERV-K Env-specific CAR-T cells in the treatment of breast cancer and the development of anti-HERV-K Env vaccines. Further efforts are urgently needed in the investigation of this promising and novel therapeutic approach.

Continuing with the focus on HERV-K Env, Weyerer et al. analysed HERV-K Env expression and function in Renal Cell Carcinoma (RCC) as well as the importance for prognosis of patients with RCC. Using a tissue microarray of 374 tumours including clinical data from RCC patients demonstrated that the highest amount of HERV-K Env protein expression and the strongest significant membrane expression were found for clear cell RCC versus other RCC subtypes. The authors also demonstrated that high HERV-K Env total protein expression of all renal tumour subtypes significantly correlated with low tumour grading and a longer disease specific survival. In addition, HERV-K Env was found to regulate proliferation and invasion depending on p53 and methylation status, underlying how epigenetic regulation of HERVs is closely related to cancer progression suggesting epigenetic therapy as a novel approach (4). Thus, this study supports a role for HERV-K Env as a single prognostic indicator for better survival of RCC and as a new tumour antigen, accessible at the membrane for possible antibody targeting.

The four Original Research and three Review articles published in this Research Topic provide new insights for the role of HERVs in cancer biology and therapy. In this landscape, HERVs have been recognized as potential cancer hallmarks, and could represent novel disease biomarkers as well as targets for therapy.

## Author Contributions

All the authors were editors of the Research Topic and equally have made substantial, direct and intellectual contribution to the work, and approved it for publication.

## Conflict of Interest

The authors declare that the research was conducted in the absence of any commercial or financial relationships that could be construed as a potential conflict of interest.

## Publisher’s Note

All claims expressed in this article are solely those of the authors and do not necessarily represent those of their affiliated organizations, or those of the publisher, the editors and the reviewers. Any product that may be evaluated in this article, or claim that may be made by its manufacturer, is not guaranteed or endorsed by the publisher.
